# Adipose tissue measurement in clinical research for obesity, type 2 diabetes and NAFLD/NASH

**DOI:** 10.1002/edm2.335

**Published:** 2022-04-06

**Authors:** Adrian Vilalta, Julio A. Gutiérrez, SuZanne Chaves, Moisés Hernández, Silvia Urbina, Marcus Hompesch

**Affiliations:** ^1^ ProSciento San Diego California USA; ^2^ Scripps Center for Organ Transplantation La Jolla California USA

**Keywords:** adipose tissue distribution, diabetes, NAFLD/NASH, obesity

## Abstract

**Introduction:**

Excess body fat is linked to higher risks for metabolic syndrome, type 2 diabetes mellitus (T2DM), and cardiovascular disease (CV), among other health conditions. However, it is not only the level but also the distribution of body fat that contributes to increased disease risks. For example, an increased level of abdominal fat, or visceral adipose tissue (VAT), is associated with a higher risk of nonalcoholic fatty liver disease (NAFLD) and nonalcoholic steatohepatitis (NASH).

**Methods:**

A review of the most relevant primary and secondary sources on body composition from the last 25 years was conducted. Relevant articles were identified using PUBMED and Google Scholar. Narrative synthesis was performed as statistical pooling was not possible due to the heterogeneous nature of the studies.

**Results:**

The body mass index (BMI) is commonly used as a proxy measure of body fatness. However, BMI does not reflect the level and distribution of body fat. Other anthropometric methods such as waist circumference measurement and waist‐hip ratio, as well as methodologies like hydro densitometry, bioelectrical impedance, and isotope dilution are also limited in their ability to determine body fat distribution.

Imaging techniques to define body composition have greatly improved performance over traditional approaches. Ultrasound (US), computed tomography (CT), dual‐energy X‐ray absorptiometry (DXA), magnetic resonance imaging (MRI), are now commonly used in clinical research. Of these, MRI can provide the most accurate and high‐resolution measure of body composition. In addition, MRI techniques are considered the best for the determination of fat at the organ level. On the other hand, imaging modalities require specialized, often expensive equipment and expert operation.

**Conclusions:**

Anthropometric methods are suitable for rapid, high‐volume screening of subjects but do not provide information on body fat distribution. Imaging techniques are more accurate but are expensive and do not lend themselves for high throughput. Therefore, successful trial strategies require a tiered approach in which subjects are first screened using anthropometric methods followed by more sophisticated modalities during the execution of the trial.

This article provides a brief description of the most clinically relevant adipose tissue measurement techniques and discusses their value in obesity, diabetes, and NAFLD/NASH clinical research.

## Graphical abstract

Excess body fat is linked to higher risks for metabolic syndrome, type 2 diabetes mellitus (T2DM) and cardiovascular disease (CV), among other health conditions. However, it is not only the level but also the distribution of body fat that contributes to increased disease risks. This review covers the current literature on the most clinically relevant body composition techniques including BMI, BIA, isotope dilution, ultrasound, DXA, CT and MRI, and discusses their value in obesity, diabetes, and NAFLD/NASH clinical trials.

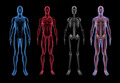

## 1 INTRODUCTION

Obesity, defined as a body mass index (BMI) equal or superior to 30 kg/m^2^, is a world‐wide epidemic with over 108 million obese children and 604 million adults in 2015.^1^, ^2^ According to the Centers for Disease Control and Prevention (CDC), in 2017 to 2018, 42.4% of adults and 19.3% of children and adolescents in the U.S. are obese.^1^ Several epidemiologic studies have shown the association between obesity and an increase in all‐cause mortality.^3^ According to some estimates, high levels of body fat as reflected by BMI is the second root cause of death and disability in the U.S. after tobacco.^4^ The obesity trends are especially concerning in the paediatric population, since about ten percent of school children world‐wide are estimated to carry excess body fat.^5^ Children who are obese have 50 to 80% chances of growing up to be obese adults.^6^

The impact of obesity on morbidity and mortality can be attributed to the links between excess body fat and an increased risk for many health conditions including type 2 diabetes (T2DM), cardiovascular disease (CV), stroke, arthritis, metabolic syndrome, nonalcoholic fatty liver disease (NAFLD) and nonalcoholic steatohepatitis (NASH).^7^, ^8^, ^9^

Evidence shows that not all excess fat contributes to disease risk in the same way. Studies have shown that abdominal fat (visceral adipose tissue; VAT) is more dangerous than subcutaneous fat because visceral fat cells release proteins that contribute to inflammation, atherosclerosis, dyslipidemia and hypertension. Consequently, VAT is more strongly associated with T2DM than other manifestations of obesity.^10^, ^11^, ^12^ Similarly, understanding fat‐related diseases and their mechanisms of action requires ever more detailed fat distribution measurements going from the whole body to body sections, to organs and tissues, and ultimately, the cellular level.

Current research and clinical practice have access to a growing number of tools to estimate body composition. The successful development of health interventions aimed at reducing the rate and health impact of obesity requires the use of the right body composition technologies for the clinical goal. On one end of the spectrum, anthropometric approaches are appropriate for rapid and cost‐effective screening of subjects, while on the other, imaging techniques can provide high‐definition adipose tissue distribution data but are expensive and their use requires a high level of technical expertise.

The present article will provide a brief overview of the most‐commonly used body composition techniques with an emphasis on the measurement of body fat, the body composition models they are based on and provide examples of their application in clinical research.

## 2 BODY COMPOSITION (BC) MODELS

When discussing body composition, it is useful to think of the human body as composed of different ‘compartments’.^13^ We will briefly discuss the differences in these models as they introduce concepts useful for the understanding of the different body composition measurement methodologies. An in‐depth discussion of BC models can be found in Müller et al.^14^

### 2.1 One‐compartment (1C) model

In the simplest approach, the body can be considered as one unit. When this model is used, the clinician will draw inferences solely from the person's weight, height, and other anthropometric measures and health risks. The most relevant method in this category is the body mass index (BMI).^15^

### 2.2 Two‐compartment (2C) model

In the two‐compartment model (2C), the body weight is divided into fat mass (FM) and fat‐free mass (FFM). The anhydrous FM is assumed to have a density of 0.9007 g/cm^3^, whereas the FFM is assumed to have a density of 1.1000 g/cm^3^ and water content of 73.72%. Hydro densitometry (HD) and air displacement plethysmography (ADP) are based on 2C model.^16^

### 2.3 Three‐compartment (3C) model

The three‐compartment (3C) model of body composition includes a third component where the FFM is divided into lean tissue mass (LTM) and bone mineral content (BMC). In the 3C model, the FFM is divided into total body water (TBW) and the remaining solids (fat‐free dry mass; FFDM). The 3C model provides more detailed information on body composition than the 2C model but must be used with caution in patients with depleted body protein or bone mineral mass, as the estimated values for density, and thus, the final estimate of body FM will not be accurate. One of the most commonly used 3C model method is dual‐energy X‐ray absorptiometry (DXA; formerly DEXA).^16^

### 2.4 Four‐compartment (4C) model

The 4C model of BC is obtained by combining many methods to partition body mass into fat, mineral, total body mass (TBM) and protein (residual), and thus, removes the need to make assumptions about the relative proportion of these constituents in the body. The 4C model controls for biological variability and it is, therefore, theoretically more valid than the 3C model.

Importantly, the 4C model is the basis for the multi‐modality reference method to assess body composition. As discussed by Lee and Gallagher, this method integrates total body water (TBW) data from deuterium dilution, bone mineral mass from dual‐energy X‐ray absorptiometry (DXA), plus body mass and volume from air displacement plethysmography (ADP).^17^

The 4C method is, however, often limited in clinical settings and large studies, in view of the time, cost and equipment needed for the multiple measurements.^16^

### 2.5 Multicompartment models

Atomic models of body composition require the direct analysis of the major elements of the body. Neutron activation analysis (NAA) can be used to measure the total body content of elements (calcium, sodium, chloride, phosphorus, nitrogen, hydrogen, oxygen and carbon). Although the multicompartment models provide accurate measures of body composition, for validating other methods, the lack of appropriate facilities, the high expense and the exposure to radiation limit their regular use.

## 3 BODY COMPOSITION TECHNIQUES

### 3.1 Body mass index (BMI) and other anthropometric‐based techniques

BMI, the most commonly used metric in this class, is defined as a person's weight in kilograms divided by the square of height in meters.^18^ Although BMI is useful as a screening tool, it does not diagnose body fatness or the health of an individual. To determine if a specific BMI is a health risk for the individual, the healthcare provider will compare against actuarial tables and perform further assessments. Such assessments include evaluations of diet, physical activity and family history, among others. Some of the known limitations of BMI are as follows:

- For a given BMI, women tend to have more body fat than men.
- For a given BMI, Blacks have less body fat than Whites, and Asians have more body fat than Whites.^19^, ^20^, ^21^
- At the same BMI, older people, on average, tend to have more body fat than younger adults.
- At the same BMI, athletes have less body fat than non‐athletes.

For children and teens, BMI is age‐ and sex‐specific and is often referred to as BMI‐for‐age. A child's weight status is different from adult BMI categories. Children's body composition varies as they age and varies between boys and girls. Therefore, BMI levels among children and teens need to be expressed relative to other children of the same age and sex.^22^ The reader is referred to the World Health Organization (WHO) webpage for comprehensive BMI‐for‐age charts and tables.^23^

### 3.2 Hydro densitometry (HD)

Hydro densitometry (HD), or under water weighing, was considered the reference method for the determination of body fat before the arrival of body imaging techniques.^24^ This technique is based on the principle whereby the volume of a body is equal to the volume of liquid displaced by it. A correction is made for the buoyancy of the air in the lungs and other body spaces. In this manner, body weight (BW) is measured in the air and water to determine body density (Db). Body fat (BF) is determined with either of the following equations:

BF = (4.57/Db–4.142) × 100^25^

BF = (4.95/Db–4.5) × 100^26^

HD has several limitations including the need for costly equipment, the assumption of specific tissue densities, which may differ across populations; residual lung volume can be a source of error, air contained within an individual's swimsuit, skin, head, and/or body hair or internally are sources of potential error too. In addition, the technique cannot measure distribution of FFM or FM. The impact of these factors on the quality of BC data using HD is explored in detail by Gibby et al. as well as Biaggi et al.^27^, ^28^ The reader is referred to the article by Brozek et al. for an in‐depth discussion of the theoretical basis of this technique.^25^

### 3.3 Whole‐body air displacement plethysmography (ADP)

Whole‐body air displacement plethysmography (ADP) uses the same basic principles as HD, but ADP is based on the displacement of air instead of water.^24^ Body fat measurements using ADP are highly correlated with those using HW, BIA and DXA across a relatively wide range of body fat levels in healthy adults. ADP is quick, comfortable and non‐invasive. However, ADP’s accuracy drops at either extreme of body fat composition as determined by DXA.^29^ Levenhagen et al. report data from a direct comparison of ADP to HD, BIA and DXA.^30^

### 3.4 Bioimpedance (BIA)

Bioimpedance analysis (BIA) is a commonly used, non‐invasive, low‐cost method to determine body fat content. Bioimpedance or bioelectrical impedance refers to the property of biological tissues to impede or resist an alternating electrical current. In BIA, the body is modeled as five cylindrical compartments; the trunk and the four limbs, while fat is an insulator. Flowing from Ohm's Law, the impedance is proportional to the height and inversely proportional to the cross‐sectional area of each compartment. A weak electric current is made to flow between two electrodes, typically on either hand or one hand and one foot. Most body water is stored in muscle. Therefore, if a person is more muscular there is a high chance that the person will also have more body water, which leads to lower impedance. Impedance is then used to estimate total body water (TBW), which can be used to estimate fat‐free body mass and, by difference with body weight, body fat.^31^

While BIA performs well at a population level in well‐controlled studies, its performance in an individual is questionable.^31^ Variations in limb length recent physical activity, nutrition, body temperature and hydration, blood chemistry, ovulation and electrode placement are potential sources of error; BIA is, therefore, not the method of choice to determine VAT.^32^

For a detailed discussion of BIA capabilities in comparison to DXA, please refer to Marra et al.^33^

### 3.5 Isotope dilution techniques

Total body water (TBW) is an important parameter in the estimation of body composition. The body of lean adults is 50%–60% water dropping to less than 40% if obese. When adequate food and drink are available, body water is in a state of flux, with water molecules constantly entering and leaving the body. However, in adults, the amount of body water remains relatively constant, changing only by a few percent.

Water is found exclusively in the FFM, which is estimated to be approximately 73.2% in adults. TBW includes both intracellular fluid and extracellular fluid. With an estimate of TBW, the amount of FFM can be calculated. Body FM is then determined as the difference between body weight and FFM.^34^, ^35^ Fat mass and FFM can be calculated using TBW, assuming that the hydration of FFM remains stable at a ratio of TBW/FFM equal to 0.73. Fat mass is calculated in the TBW method as body weight devoid of FFM. The hydration of FFM although regarded constant at 0.73 may be influenced by several health factors thus limiting its use for quantification of excess fluid.^36^

Water containing either hydrogen (deuterium,^2^H; tritium ^3^H) or oxygen (^18^O) isotopes can be used to estimate TBW by dilution. Stable, that is, non‐radioactive, isotopes such as deuterium have been used in human studies for over 50 years. We refer the reader to the study published by Schoeller et al. on the accuracy of the TBW determination using the stable isotopes^2^H and ^18^O.^37^ Body water can be sampled in the form of saliva, urine, plasma or human milk, and the enrichment of stable isotopes such as deuterium can be measured by a number of analytical methods such as mass spectrometry or Fourier‐transformed infrared spectrometry.^34^, ^35^

The reader is referred to publications 1450 and 1451 from the International Atomic Agency (IAEA) for an in‐depth discussion of the use of deuterium dilution techniques in body composition assessment.^34^, ^35^

### 3.6 Body composition through imaging techniques

#### 3.6.1 Ultrasound (US)

Ultrasonography is an attractive imaging option for a first‐line diagnostic method to evaluate large groups of patients for clinical studies due to its relative speed and non‐invasive nature. US is capable of directly measuring VAT, in addition to subcutaneous adipose tissue (SAT) in different sections of the abdomen.^38^, ^39^ US techniques have been used to show skin‐subcutaneous fat boundaries as well as fat‐muscle and muscle‐bone interfaces.^38^ On the contrary, there is considerable variability in US methods, differing in US frequencies and measurement sites. This variability leads to some difficulty when comparing findings in the literature.^38^

The reader is referred to Ponti et al. for an in‐depth discussion of US methodologies, technical considerations and the clinical value of US in body composition studies.^40^

#### 3.6.2 Ultrasound and NAFLD/NASH

Healthy liver tissue is as echogenic as adjacent organs such as the spleen and kidneys. However, when there is an abnormal retention of fat in the liver (steatosis), the organ appears brighter in the ultrasound image due to increased scatter of the ultrasound beam by fat droplets. Similarly, liver fat weakens the ultrasound beam, resulting in blurry imaging of liver structures such as intrahepatic vessels and bile ducts.^41^ Ultrasound can be used to both diagnose and grade the degree of liver steatosis. Liver brightness on the ultrasound image is compared to that of the kidney or spleen which work as internal standards. However, ultrasound techniques are relatively insensitive for the detection of mild steatosis and may not perform adequately if there is another underlying liver disease.

Ultrasound is a safe, widely available and patient friendly imaging modality. The associated cost of ultrasound is low compared to other imaging modalities. On the contrary, there are some limitations of the technology including (a) overestimation of steatosis in heavy set subjects and (b) confounding of the ultrasound image by inflammation, fibrosis and other features of chronic hepatic disease.^42^ In addition, the quality of the ultrasound diagnosis is strongly dependent of the operator skills, calibration of the instrument and manufacturer of the machine. Given these performance characteristics, ultrasound produces qualitative classifications of steatosis that are hard to compare between subjects and clinical sites. However, new developments in this field may overcome its current limitations of US.^43^, ^44^, ^45^

Newer, quantitative US modalities are reported to have superior performance compared with semi‐quantitative US.^46^ The controlled attenuation parameter (CAP) is increasingly being used to estimate liver fat. A CAP value can be obtained simultaneously with a liver stiffness measurement (LSM) by vibration‐controlled elastography (VCTE) commercially known as FibroScan. The article by Ajmera and Loomba provides a detailed discussion of US and the value of the CAP parameter in the assessment of liver fat.^47^

#### 3.6.3 Computed tomography (CT)

CT uses computer processing of X‐ray data of the body to produce a high‐resolution, 3‐dimensional image. The differences in X‐ray attenuation by different body fat and lean tissues are used in CT to calculate differences in composition and location in the body. CT has been used to determine fat in liver and skeletal muscle.^48^, ^49^ Although in principle CT could be used to estimate organ and body part volumes, in practice CT is used to analyse 2‐dimensional slices of the body. This limitation is due in part to the need to minimize exposure of the subject to ionizing radiation (X‐rays). This is particularly relevant in clinical trials where healthy volunteers are involved.

Gibby et al. report data from a direct comparison of BC using CT and HD and ADP.^27^ Their study showed high correlation between the ADP and CT data using two methods (Schneider method, *r* = 0.9806 and Beam method, *r* = 0.9804).

### 3.7 CT and NAFLD/NASH

CT images are created from detection of X‐rays traversing tissues. Weakening of the X‐ray as it passes through the body is a key parameter used to define the brightness of the tissue in the CT image. In this manner, dense tissues will attenuate the X‐ray beam the most and result in a brighter rendition on the image. A healthy liver will appear brighter than the spleen in a CT scan. As fat content in the liver increases, its corresponding image will become darker.^50^

CT provides fast data acquisition and quantitative results. On the contrary, similarly to US, CT is insensitive in cases of mild steatosis. CT liver images can also be confounded by other factors such as concentration of iron, glycogen, and hematocrit.^51^ Furthermore, there is a strong dependence on scanner‐specific calibration which depends on the instrument manufacturer and the underlying calibration algorithms.^52^ CT is not usually recommended as the primary modality to measure liver fat given its lack of sensitivity for mild steatosis and the need for exposure of the subjects to ionizing radiation (X‐rays).

#### 3.7.1 Dual‐energy X‐ray absorptiometry (DXA)

Use of DXA yields body fat percentage, body composition and bone mineral density. DXA is based on the use of two low energy X‐ray beams. The attenuation of X‐rays as they pass through the body is dependent on the thickness of the tissue and the tissue's attenuation coefficient, which dependents on the X‐ray energy. Comparing the attenuation for each of the two X‐ray energies, DXA provides a detailed image of the body.^53^ DXA is the most widely used method to determine bone density where it is considered the reference method. DXA can also be used to measure total body composition, fat content and distribution.^54^

DXA is thought to be more accurate than body density‐based methods for estimating total body fat.^55^ A potential source of error is that the DXA analysis assumes a constant hydration of lean soft tissue.

The reader is referred to in‐depth discussions of the applications and technical aspects of DXA by Bazzocchi et al. and Marra et al.^33^, ^56^ An assessment of the value of DXA to assess body composition in athletes and active people can be found in Nana et al.^57^

#### 3.7.2 Magnetic resonance imaging (MRI)

Magnetic resonance imaging (MRI) technologies allow for the precise measurement of body fat and other soft tissues such as muscle using the magnetic properties of chemical elements.^58^, ^59^ Quantitative fat water imaging MRI, a commonly used imaging method, has been used to generate precise measurements of lean tissue and body fat. This approach is based on the different magnetic resonance frequencies of protons in fat and water; these differences are used for separating the two signals into a fat image and a water image.

Importantly, a magnetic resonance image on its own is not calibrated to be quantitative. Two MRI techniques that successfully address this limitation are proton density fat fraction (PDFF) measuring the fraction of fat in MR‐visible soft tissue and fat‐referenced MRI.^60^, ^61^

### 3.8 MRI and NAFLD/NASH

Current MRI imaging technologies are considered the reference method for the measurement of liver steatosis. In contrast with CT and US, MRI can directly measure and distinguish the signal from water versus triglycerides. Current MRI algorithms can quantitate liver fat. This methodology is commonly referred as MRI proton density fat fraction imaging or MRI‐PDFF for short.

Data from NAFLD studies using MRI‐PDFF show that the findings are highly reproducible across scanners.^62^ There is also high correlation between MRI‐PDFF liver fat measurements and biochemical determination of triglycerides.^63^ Importantly, MRI‐PDFF accurately classifies using histology as a reference method, and the change in PDFF accurately classifies change in steatosis over time.^64^

The power of MRI‐PDFF has been used to evaluate liver fat‐reducing drug candidates. For example, Loomba et al. reported that obeticholic acid (OCA) was better than placebo in reducing liver fat. Data from this multicentre trial showed the association between a 30% decline in MRI‐PDFF relative to baseline and histologic response in NASH.^65^ In another example, Beysen et al. explored the therapeutic potential of drug candidate FT‐4101, a fatty acid synthase (FASN) inhibitor, on hepatic steatosis in patients with NAFLD. The authors used MRI‐PDFF to measure the impact of the drug on liver fat.^66^

### 3.9 Comparison of capabilities

Table 1 summarizes the capabilities of different techniques of body composition analysis.

**TABLE 1:** Body composition techniques‐Summary of capabilities

	ADP	BIA	CT	DXA	MRI	US	ID
Total Fat	Yes	Yes	Yes	Yes	Yes	No	Yes
Total Lean Tissue	Yes	Yes	Yes	Yes	Yes	Approx	Yes
VAT	No	No	Yes	Approx	Yes	Approx	No
Muscle Volume	No	No	Yes	No	Yes	No	No
Diffuse Fat Infiltration	No	No	Yes	No	Yes	Approx	No
Ionizing Radiation	No	No	Yes	Yes	Yes	No	Depends

Abbreviations: ADP, Air displacement plethysmography; BIA, bioimpedance analysis; CT, computed tomography; DXA, dual‐energy X‐ray absorptiometry; ID, isotope dilution; MRI, magnetic resonance imaging; US, ultrasound; VAT, visceral adipose tissue.

## 4 ADDITIONAL EXAMPLES OF THE USE OF BODY COMPOSITION MODALITIES IN CLINICAL RESEARCH

Development of novel clinical interventions for obesity, diabetes, metabolic syndrome, and nonalcoholic liver disease (NAFLD) and nonalcoholic steatohepatitis (NASH) depend on the accurate and reproducible determination of body fat. In this section, we will discuss the value of body composition testing modalities and provide a few examples of their application in clinical research.

### 4.1 Obesity and diabetes

Anthropometric body composition techniques have been extensively used in obesity and diabetes studies for decades. In fact, BMI is used as the key metric defining overweight and obesity and the association of high BMI and the risk for type 2 diabetes mellitus (T2DM) is well established.^1^, ^67^ However, as discussed above, BMI as a standalone metric has limited value in clinical research. On the contrary, some recent studies highlight the power of using a hybrid modality approach, that is, where more than one body composition modality is used. As a case in point, Mavros et al. studied the impact of exercise and the resulting changes in body composition, insulin resistance and HbA_1c_ in adults with T2DM.^68^ These authors used a combination of anthropometric measures, bioelectrical impedance (BIA) and computed tomography (CT) to show the positive impact of high‐intensity progressive resistance training on the subjects’ metabolic health.

Rowan et al. reported findings on the value of metformin to manage gestational diabetes.^69^ They used a tiered approach in which the body composition of both the mother and child were evaluated first using anthropometrics and then followed up by a DXA scan. These authors concluded that children exposed to metformin had larger measures of subcutaneous fat, but overall body fat was the same as in children whose mothers were treated with insulin alone. They suggested that further follow‐up will be required to examine whether these findings persist later life and whether children exposed to metformin will develop less visceral fat and be more insulin sensitive.

An interesting example of the use of body composition endpoints in the evaluation of an antidiabetic drug is provided by Kamei et al.^70^ These authors report the impact of tofogliflozin, a sodium‐glucose cotransporter 2 inhibitor (SGLT2), on glycaemic control and body composition. Kamei and co‐workers relied on BIA to assess the body composition changes in their subjects. The authors showed that tofogliflozin exerted beneficial effects on metabolic parameters such as body weight, HbA_1c_ and uric acid without severe adverse effects. In addition, they showed that body fat mass was decreased accompanied by the reduction of body water as well as skeletal muscle mass.

Kigaru et al. published a compelling example of the use of stable isotope dilution techniques (SIDT) to assess the level of body fat in 179 children aged 8 to 11 years in Kenya.^71^ Their goal was to evaluate BMI‐for‐age scores (BAZ) as compared to SIDT, the reference method for body composition. The participants were given a 2H dose based on their weight as per IAEA guidelines.^34^, ^35^ Saliva samples were collected at 2 and 3 h post‐dosing. The authors reported that prevalence of adiposity by reference SIDT (24·0%) was significantly higher than that of obesity by BAZ >2 SD (2·8%). Only 11·6% of children with excess body fat were correctly diagnosed as obese by BAZ >2 SD. The use of BAZ >1 SD for overweight and obesity showed fair concordance coefficient (κ = 0·409, *p* < ·001) with 32·5% of children with excess fat positively identified as overweight and obese. The authors concluded that WHO BMI‐for‐age cut‐off points severely underestimate the prevalence of overweight and obesity compared with body composition assessment by stable isotope dilution techniques.

## 5 CONCLUSIONS

Clinical scientists have access to a wide range of techniques to estimate body fat and its distribution in the body. Anthropometric approaches like BMI are suitable for rapid, high‐volume screening of subjects but do not provide information on body fat distribution. On the other end of the spectrum, imaging techniques are more accurate but are expensive and do not lend themselves for high throughput. Evaluation of the effectiveness of drug candidates and other health interventions requires the use of the right testing technologies balancing cost, throughput and data quality. Therefore, successful trial strategies may require a tiered approach in which subjects are first screened using anthropometric methods followed by more sophisticated modalities during the execution of the trial. Good examples of this approach can be found in the development of drugs for NAFLD/NASH, in which subjects at risk are first screened using BMI and/or ultrasound modalities, followed by MRI‐PDFF imaging for the evaluation of the effectiveness of the drug. Another key factor to consider is data harmonization, especially when collecting clinical data at multiple clinical sites. Careful selection of the body composition testing modalities to use and cross‐validation of the testing platforms is essential for the successful execution of clinical trials.

## CONFLICT OF INTEREST

All authors are employees of ProSciento.

## AUTHOR CONTRIBUTION

**ADRIAN VILALTA:** Conceptualization (lead); Data curation (lead); Writing – original draft (equal); Writing – review & editing (equal). **Julio Gutierrez:** Writing – review & editing (equal). **Moises Hernández:** Writing – review & editing (supporting). **SuZanne Chaves:** Writing – review & editing (supporting). **Silvia Urbina:** Writing – review & editing (supporting). **Marcus Hompesch:** Resources (lead); Visualization (supporting); Writing – review & editing (equal).

## Data Availability

N/A
